# Anterior segment dysgenesis after overexpression of transforming growth factor-β-induced gene, βigh3, in the mouse eye

**Published:** 2007-10-16

**Authors:** Jung-Eun Kim, Min-Su Han, Yong-Chul Bae, Hong-Kyun Kim, Tae-Im Kim, Eung Kweon Kim, In-San Kim

**Affiliations:** 1 Department of Molecular Medicine, Kyungpook National University School of Medicine,; 2 Cell and Matrix Research Institute, Department of Biochemistry and Cell Biology, Kyungpook National University School of Medicine, Daegu, 700-422, Korea,; 3 Department of Oral Anatomy, Kyungpook National University School of Dentistry, Daegu, 700-412, Korea,; 4 Department of Ophthalmology, Kyungpook National University School of Medicine, Daegu, 700-422, Korea,; 5 Corneal Dystrophy Research Institute, Department of Ophthalmology, Yonsei University College of Medicine,; 6 BK21 Project Team of Nanobiomaterials for Cell-based Implants, Yonsei University, Seoul, 120-752, Korea

## Abstract

**Purpose:**

βigh3 is a transforming growth factor-β-inducible cell adhesion molecule and its mutations are responsible for human autosomal dominant corneal dystrophies. Previously, we have studied the molecular properties of βigh3 in vitro and reported that βigh3 polymerizes to form a fibrillar structure and interacts with several extracellular matrix proteins including type I collagen. This study aimed to understand the role of elevated circulating levels of normal βigh3 in eye development and corneal diseases.

**Methods:**

We generated Alb-hßigh3 transgenic mice that have liver-specific expression of human βigh3 (hßigh3) under the control of the albumin (Alb) enhancer/promoter and investigated the influence of βigh3 overexpression in mouse eye. Polymerase chain reaction (PCR) genotyping, western blotting, and ELISA were performed to generate Alb-hßigh3 transgenic mouse lines. To identify the ocular pathology, electron microscopy and histological staining were employed in Alb-hßigh3 transgenic mice and wild-type mice.

**Results:**

Normal hßigh3 was ectopically overexpressed in the liver, secreted into blood stream, and reached the cornea of Alb-hßigh3 transgenic mice. Among transgenic mice, some mice had anterior segment defects including corneal opacity, disorganization of the collagen layers in the corneal stroma, and corneolenticular adhesion.

**Conclusions:**

These results suggest that βigh3 may be involved in anterior segment morphogenesis and eye development in mice. In addition, this indicates that the level of normal βigh3 expression must be properly maintained during ocular development. The phenotype observed in Alb-hßigh3 transgenic mice is similar to human eye disorders such as anterior segment dysgenesis and Peters' anomaly. Thus, this model provides a very useful tool to study human eye diseases and the control of proliferation and differentiation of neural crest-originated cells.

## Introduction

βigh3 (keratoepithelin), also known as TGFβ1, is a transforming growth factor-β (TGF-β)-induced extracellular matrix (ECM) protein that was first identified in human adenocarcinoma cells [1]. βigh3 is strongly induced by TGF-β in several cell lines including human epithelial cells, keratinocytes, and fibroblasts [2,3]. βigh3 is ubiquitously expressed in many normal human tissues such as the heart, liver, pancreas, and skin, suggesting that it may have an important function throughout the body [1]. It has been reported that βigh3 is not only expressed in the cornea of the normal human eye but also in healing corneal wounds [4-6]. During mouse development, expression of βigh3 in the cornea begins around embryonic day 15.5 (E15.5) and is sustained until E18.5 with localization in the corneal epithelium and stroma [7].

Mutations of βigh3 are responsible for 5q31-linked human autosomal dominant corneal dystrophies such as granular corneal dystrophy (GCD), Reis-Bückler corneal dystrophy (RBCD), lattice corneal dystrophy (LCD) type I and IIIA, and Avellino corneal dystrophy (ACD) [8,9]. These diseases are most often characterized by progressive accumulation of deposits in the cornea, resulting in a loss of transparency and severe visual impairment. Although mutations of βigh3 are well described in corneal dystrophy, the function of normal βigh3 in the eye is not well known. We recently reported that normal βigh3 mediates human corneal epithelial cell adhesion through α3β1 integrin [10] and that βigh3 and its mutants polymerize to form a fibrillar structure and interact with type I collagen, laminin, and fibronectin [11].

TGF-β is a multifunctional cytokine that regulates cell growth and differentiation as TGF-β inhibits epithelial cell proliferation and stimulates the proliferation of smooth muscle cells and skin fibroblasts [12,13]. TGF-β and its receptors are localized in the human anterior segment of the eye, including the cornea, and may regulate various pathophysiological responses in the anterior segment by controlling cell proliferation, differentiation, and ECM composition [14,15]. Moreover, TGF-β highly induces βigh3, which associates with ECM molecules. Based on the expression of TGF-β and βigh3 in the eye and their correlation, βigh3 as well as TGF-β may be key molecules in the pathogenesis of ocular disorders or in the eye development.

In this study, to characterize the role of βigh3 responses in ocular development, we generated transgenic mice that have liver-specific expression of normal βigh3 under the control of the albumin (Alb) enhancer/promoter [16] and we investigated in the eyes of these mice the influence of overexpressed normal βigh3 secreted from the liver. The data from this study showed that the elevated levels of normal βigh3 caused corneal opacity and anterior segment dysgenesis. Therefore, these results had particular relevance for human fetal conditions characterized by ocular abnormalities such as anterior segment mesenchymal dysgenesis. The results established an experimental model in which overexpression of normal βigh3 resulted in the gross ocular pathophysiological characteristics of these conditions.

## Methods

### Animals

All procedures concerning animal experiments in the present study were conducted according to the guidelines of Kyungpook National University. All mice were maintained on 12 h light/dark cycles in specific pathogen free (SPF) conditions and fed a sterilized standard diet.

### Construction of the Alb-hßigh3 transgene and generation of transgenic mice

To generate an Alb-hßigh3 transgene, a *Sal*I/*Xho*I fragment of a full-length human βigh3 (hßigh3, amino acids 1–683), which has high identity with mouse βigh3, was first cloned into the *Sal*I site of a plasmid that has a 4.3 kb fragment of IRES-LacZ-mp1 intron/polyA. After insertion of hßigh3, a 2.3 kb fragment of the albumin (Alb) enhancer/promoter (kindly provided by Dr. R. Palmiter, Seattle, WA) [16], which is essential for expression in the liver, was cloned into the *Not*I/*Sal*I site at the 5′-end of hßigh3-IRES-LacZ-mp1 intron/polyA. The entire construct was cut with *Not*I and *Nru*I and the 8.8 kb transgene named Alb-hßigh3 was purified. Purified Alb-βigh3 transgene was injected into the pronucleus of fertilized eggs of C57BL/6 embryos by Macrogen Inc. (Seoul, Korea). Two hundred animals were screened and seven founders were identified that exhibited integration of the Alb-hßigh3 transgene by polymerase chain reaction (PCR) genotyping. Transgenic founders were backcrossed to C57BL/6 or intercrossed. The transgenic mice and their wild-type control littermates were maintained under standard temperature and lighting.

### Identification of transgenic mice by genotype analysis

The transgene in Alb-hßigh3 founders and offspring was identified by PCR analysis of genomic DNA obtained from tail biopsies. PCR was performed in 30 μl reaction mixtures, each containing 100 ng genomic DNA, 0.2 μM each primer set, 1 mM dNTP mixture, 3 μl of 10X Taq buffer, and 1 unit of Taq polymerase. The primers were specific for hßigh3 cDNA (forward: 5′-TCA TCG ATA AGG TCA TCT CC-3′, reverse: 5′-CGG TTC AAA GTC TCA CTA GG-3′) and LacZ cDNA (forward: 5′-TAA TCA CGA CGC GCT GTA TC-3′, reverse: 5′-CGG ATA AAC GGA ACT GGA AA-3′) to amplify a 202 bp and 500 bp fragment, respectively. Amplification was performed for 35 cycles in the following PCR conditions: one min at 95 °C, one min at 48 °C for hßigh3 and at 58 °C for LacZ, and one min at 72 °C using a GeneAmp PCR System 9600 (PE Applied Biosystems, Foster City, CA). PCR products were then separated electrophoretically on 1% agarose gels and visualized after ethidium bromide staining. Primers for glyceraldehyde-3-phosphate dehydrogenase (GAPDH; forward: 5′-TGA AGG TCG GTG TGA ACG ATT TGG C-3′, reverse: 5′-CAT GTA GGC C-AT GAG GTC CAC CAC-3′) were used as a PCR control.

### Western blotting for human βigh3 in the liver

Liver and eye from wild-type and transgenic mice was prepared in radioimmunoprecipitation assay (RIPA) lysis buffer including 150 mM NaCl, 10 mM Tris pH 7.2, 0.1% SDS, 1% Triton X-100, 1% Deoxycholate, and 5 mM EDTA. Each sample was mixed with 2X sample buffer (100 mM Tris-HCl, pH 6.8, 200 mM dithiothreitol, 4% SDS, 0.2% bromophenol blue, and 20% glycerol) and boiled for 10 min. Then, the samples were separated by 10% sodium dodecyl sulfate-PAGE and transferred to nitrocellulose membrane (GE Healthcare, Buckinghamshire, UK). Blocking was performed with 5% nonfat milk in phosphate-buffered saline (PBS) for 1 h at room temperature (RT). The membrane was incubated for 2 h at RT with anti-human βigh3 antibody (diluted 1:1000 in PBS), and then reacted for 1 h at RT with peroxidase-conjugated anti-rabbit IgG antibody (diluted 1:3000 in PBS; Santa Cruz Biotechnology, Santa Cruz, CA). The blot was developed with Super Signal West Pico Chemiluminescent Substrate (Pierce, Rockford, IL). Anti-β-actin antibody was used as a control on the same filter after deprobing.

### Immunohistochemistry of human βigh3 in the liver and eye

To investigate transgenic expression, immunohistochemical staining of the mouse liver and eye was performed as described previously [17]. Briefly, sections were deparaffinized, rehydrated, and blocked by incubation in 10% H_2_O_2_ before blotting. The sections were then put in 1 mM Tris solution (pH 9.0) supplemented with 0.5 mM EGTA and heated in a microwave for 10 min to reveal the antigens. After blocking in PBS, supplemented with 1% BSA, 0.05% saponin, and 0.2% gelatin, sections were incubated overnight at 4 °C with anti-human βig-h3 antiserum in a humidified chamber. Sections were then washed three times and incubated with horseradish peroxidase-conjugated goat anti-rabbit immunoglobulins (DAKO, Glostrup, Denmark) for 90 min at RT. The signal was visualized by incubating the sections with liquid diaminobenzidine tetrahydrochloride (DAB) Chromogen (DAKO). Hematoxylin staining was used to counterstain sections.

### Enzyme-linked immunoSorbent assay of human βig-h3 in blood

The level of human βigh3 in mouse plasma was measured by enzyme-linked immunosorbent assay (ELISA; Regen Biotech, Seoul, Korea). Blood was collected through the saphenous vein into potassium EDTA-coated Microvette tubes (Sarstedt, Nümbrecht, Germany). Recombinant human βigh3 proteins and the anti-human βigh3 antibody for ELISA were prepared as described previously [10]. For ELISA, 96 well plastic flat microtiter plates (Corning, Lowell, MA) were coated overnight at 4 °C with wild-type human βigh3 protein in 20 mM carbonate-bicarbonate buffer (pH 9.6) with 0.02% sodium azide. The coated plates were then washed with PBS with 0.05% Tween-20 (PBS-T). Mouse plasma samples were diluted in PBS-T and preincubated with anti-human βigh3 antibodies in 96 well plastic round microtiter plates for 90 min at 37 °C. The preincubated samples were then transferred to the precoated plates and incubated for 30 min at RT. Thereafter, the samples were incubated with the peroxidase-conjugated anti-rabbit IgG antibodies (Santa Cruz Biotechnology) for 90 min at RT, and the plates were washed as before. The assay was developed with a substrate solution of 0.1 mg/ml o-phenylenediamine and 0.003% H_2_O_2_ for 60 min at RT in the dark. After stopping the reaction with 8 N H_2_SO_4_, the absorbance was read at 492 nm in a Bio-Rad model 550 microplate reader. The paired *t*-test was used to determine statistical significance with p<0.05 considered to denote statistical significance. Values were expressed as mean±SD.

### Gross pathology and ocular stereology

To analyze the gross appearance of the eye, enucleated mouse eyeball samples were examined under a slit lamp biomicroscope (Haag-Streit, Bern-Koeniz, Switzerland) equipped with a digital camera; images were captured with the eye image capture system (eMedio®Inc., Seoul, Korea). To capture cataracts, pupils were dilated with a drop of 1% Mydriacyl (Alcon Laboratories, Hemel Hempstead, UK) for 20–30 min while the animals were under anesthesia. The eyeball sizes were observed under a stereomicroscope with a ruler.

### Light microscopy

For the histological analysis of the cornea, enucleated mouse eyeballs were fixed in 4% paraformaldehyde (PFA) in PBS for 16 h at 4 °C, dehydrated in a graded series of ethanol, and then embedded in paraffin. Serial sections, including anterior segment defects in the eyes of Alb-hßigh3 transgenic mice, were cut at 4 μm thickness and stained with hematoxylin and eosin (H&E) or with Masson's trichrome. Masson's trichrome stained collagen fibers blue and most other intracellular and extracellular proteins red.

### Electron microscopy

Corneas were fixed in 2.5% glutaraldehyde in 0.1 M phosphate buffer (PB, pH 7.4) for 2–4 h at 4 °C. The central portion of the cornea, including the opaque region, in Alb-hßigh3 transgenic mice was removed, washed three times in 0.1 M PB, and post-fixed in 1% osmium tetroxide in 0.1 M PB for 2 h at 4 °C. After rinsing with 0.1 M PB, the samples were dehydrated in a graded series of ethanol, immersed in propylene oxide, infiltrated with an Epon mixture, embedded in a Beem capsule, and polymerized for three days. Serial ultrathin sections were cut with an ultramicrotome, collected on Formvar film-coated single slot nickel grids, and counterstained with uranyl acetate and lead citrate. The grids were examined on a Hitachi H6000 electron microscope at 80 kV accelerating voltage.

## Results

### Transgenic mice overexpressing normal hßigh3 in the liver and blood

**Figure 1:**
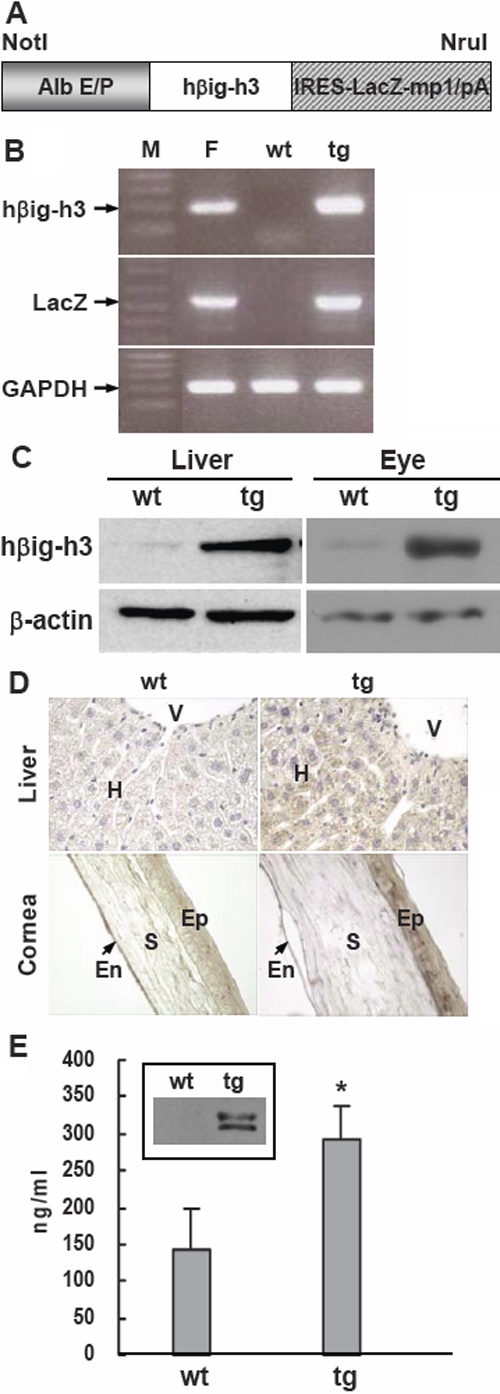
Generation of Alb-hßigh3 transgene

The mouse albumin (Alb) enhancer/promoter was used to generate transgenic mice expressing human βigh3 (hßigh3) in the liver with increased levels of hßigh3 in the blood. The full-length hßigh3 was inserted into the plasmid with Alb enhancer/promoter, followed by IRES-LacZ-mp1 intron/polyA to detect β-galactosidase activity in the liver (Figure 1A). This *Not*I/*Nru*I transgene fragment was injected into fertilized eggs of C57BL/6 and then 200 animals from injected eggs were screened to select transgenic founders. Seven founder mice were identified and three of these lines showed high expression of the transgene. To maintain the lines, they were crossed with C57BL/6 and the offspring were identified by PCR genotyping of hßigh3 and LacZ gene 10 days after birth (Figure 1B). PCR results showed 202 bp and 500 bp PCR products for hßigh3 and LacZ, respectively. To investigate ectopic expression of the hßigh3 transgene, western blotting and immunohistochemistry were performed in the liver and eye of two-month-old mice using anti-human βigh3 antibody. High expression of hßigh3 was detected by western blot analysis in the liver and in eye extracts of transgenic mice (Figure 1C). Transgenic mice also showed strong expression of the hßigh3 transgene in liver hepatocytes and in corneal epithelium by immunostaining (Figure 1D). Figure 1B-D show representative data for hßigh3 transgene expression in 28 transgenic mice. ELISA was completed for hßigh3 in plasma and the level of hßigh3 in 28 transgenic mice was 1.5–2 fold higher than in 19 wild-type mice (Figure 1E). A high level of hßigh3 expression was also observed by western blot analysis in the plasma of transgenic mice (Figure 1E). As the human βigh3 antibody partially cross-reacted with both mouse and human βigh3, the amount in wild-type mice likely represented the level of endogenous mouse βigh3 in tissue and blood. Finally, we generated Alb-hßigh3 transgenic mice that overexpress hßigh3 under the control of the Alb enhancer/promoter. Stable transgenic mice were continuously propagated in the C57BL/6 background to maintain the lines and to analyze the phenotype.

**A**: The diagram of Alb-hßigh3 transgene shows the albumin enhancer/promoter (Alb E/P), the human βigh3 (hßigh3), and IRES-LacZ-mp1 intron/polyA (IRES-LacZ-mp1/pA). *Not*I and *Nru*I indicate enzyme sites in the transgene for pronuclear injection. **B**: PCR genotyping for hßigh3 and LacZ gene in wild-type (wt) and transgenic (tg) mice. The 202-bp hßigh3 and 500 bp LacZ amplification products were produced by PCR in Alb-hßigh3 transgenic mice. PCR for glyceraldehyde-3-phosphate dehydrogenase (GAPDH) was performed as a control. M and F indicate a DNA size marker and a founder mouse as a positive control for PCR, respectively. **C**: western blot analysis of hßigh3 expression in liver and eye extracts from wild-type (wt) and transgenic (tg) mice. The hßigh3 protein was highly expressed in the transgenic mouse compared to the wild-type mouse. β-Actin was used as an internal protein control. **D**: Immunohistochemical study of wild-type (wt) and transgenic (tg) liver and cornea at two months of age using anti-human βigh3 antibody. Expression of hßigh3 transgene was strongly detected in liver hepatocytes and corneal epithelium of Alb-hßigh3 transgenic mouse. Abbreviations: H, hepatocyte; V, terminal hepatic venule; Ep, corneal epithelium; S, corneal stroma; En, corneal endothelium. **E**: Human βigh3 levels in plasma of wild-type (wt) and transgenic (tg) mice. ELISA showed that hßigh3 levels in plasma were elevated twofold in transgenic mice. The plasma levels show the average for 19 wild-type mice and 28 transgenic mice. High expression of hßigh3 protein was detected in plasma of transgenic mice by western blotting. The asterisk indicates a statistically significant difference (p<0.001) calculated by paired *t*-test. The western blotting and ELISA data are representative of three independent experiments.

### Gross phenotype and electron microscopic examination of the mice eyes with hßigh3 overexpression

**Figure 2:**
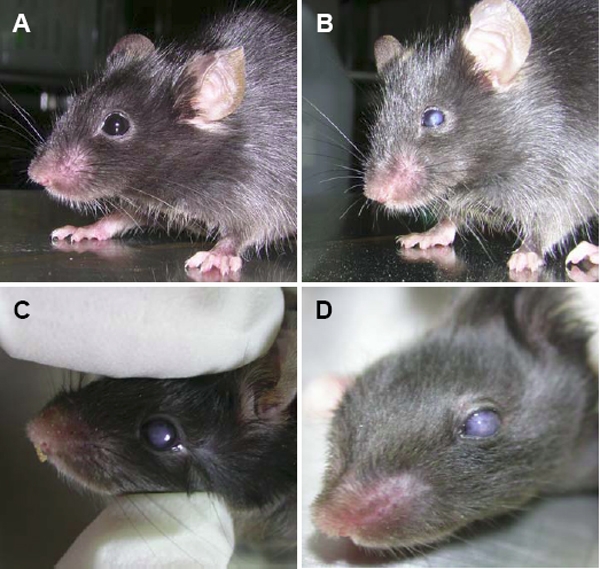
Gross appearance of wild-type and Alb-hßigh3 transgenic mouse

**Figure 3:**
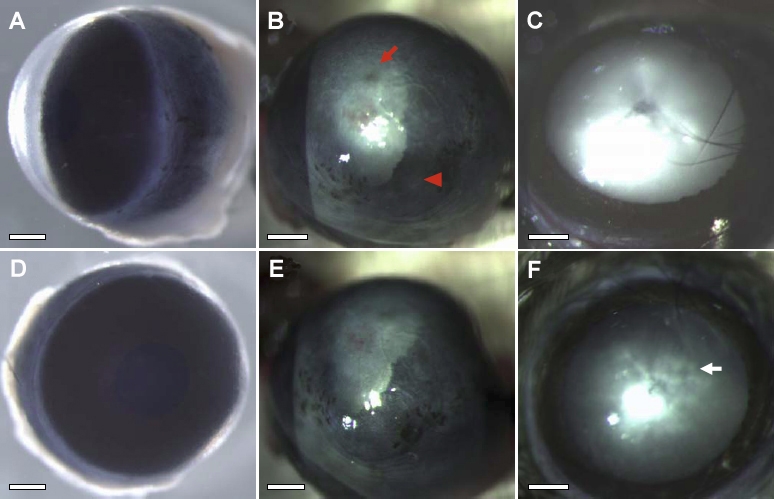
Phenotypes in two-month-old Alb-hßigh3 transgenic mouse eye

**Figure 4:**
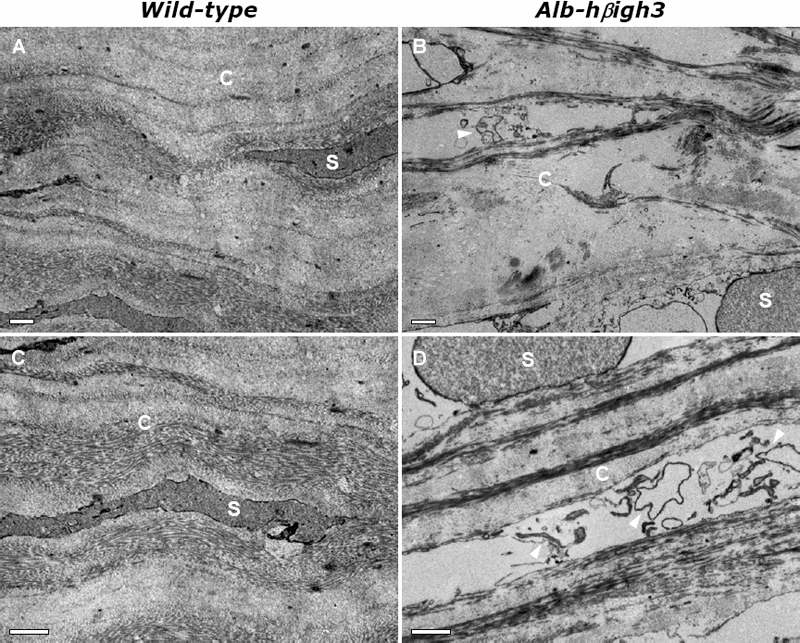
Electron micrographs showing stroma of central portion of cornea in wild-type and Alb-hßigh3 transgenic mice

Among the Alb-hßigh3 transgenic mice identified by PCR genotyping 10 days after birth, eight transgenic mice displayed a central corneal opacity, a characteristic phenotype that was visible when the eyelids opened around two weeks after birth. At the age of two months, when plasma could safely be collected, the level of hßigh3 was measured in all wild-type and transgenic mice to establish hßigh3 overexpression in plasma. The average bodyweight and eye size were identical for wild-type (22±0.6 g, 3.5±0.05 mm) and transgenic mice (22±0.4 g, 3.5±0.08 mm) at two months of age. Internal organs such as the liver and kidney were phenotypically normal (data not shown) and there was no specific phenotype, even in the liver where ectopic normal βigh3 was produced under control of the albumin promoter (Figure 1D). This was performed through systematic sampling and measurements in all wild-type and transgenic mice. Even though the bodyweight and the size of eyes were no different between wild-type and transgenic mice, various phenotypes with diverse defect sizes were investigated in the defective eye of transgenic mice that had a corneal opacity (Figure 2). The gross ocular phenotypes of Alb-hßigh3 transgenic mice showed corneal opacification (Figure 3B). The opacity sometimes accompanied a cataract, which was investigated in the eye of transgenic mice after pupil dilatation (Figure 3C). These anomalies were never seen in normal eyes of wild-type mice (Figure 3A). Corneal opacity was observed bilaterally with cataracts in Alb-hßigh3 transgenic mice with eye defects. Among eight Alb-hßigh3 transgenic mice with corneal opacity, bilateral defects with cataracts were observed in five mice and unilateral defect was shown in the remaining three mice. Electron microscopy showed that the collagen fibers and fibrils were disorganized with an irregular arrangement in the corneal stroma of the defected eye in transgenic mice compared to the stroma in wild-type mice, which were compact and well organized (Figure 4A). Additionally, tissue debris was frequently observed in the spaces among the disorganized collagen fibers (Figure 4B).

The eye of a two-month-old transgenic mouse (**B**) had a corneal opacity with a characteristic phenotype compared to wild-type (**A**). **C** and **D** show the left eyes with corneal opacity in one-year-old Alb-hßigh3 transgenic mice.

**A** and **D**: Normal eye of a wild-type mouse. **A** and **D** are a lateral view and a frontal view of the eye with transparent cornea, respectively. **B** and **E**: Mutant eye with corneal opacity in Alb-hßigh3 transgenic mouse. **B** and **E** show different angle views of the defective eye in the transgenic mouse. The Red arrow indicates the corneal opacification and the red arrowhead indicates the relatively clear zone in the transgenic mouse. **C** and **F**: The mutant eye with cataract in Alb-hßigh3 transgenic mouse. **C** and **F** show different angle views of the eye with cataract in the transgenic mouse. The white arrow indicates cortical opacity of the lens. The scale bars are equal to 0.5 mm.

Corneas from mice were fixed in 2.5% glutaraldehyde and subjected to electron microscopy. **A** and **C**: In wild-type mice, collagen fibers and fibrils were regularly and compactly arranged parallel to the epithelial surface. However, in Alb-hßigh3 transgenic mice (**B** and **D**), the collagen fibers and fibrils were disorganized. Tissue debris was occasionally observed between the collagen fibers (arrowhead). **A** and **B** are images corresponding to 5K and **C** and **D** are images corresponding to 8K. The scale bars are equal to 2 μm. Abbreviations: C, collagen layers; S, stromal cells.

### Histological analysis of the anterior segment

**Figure 5:**
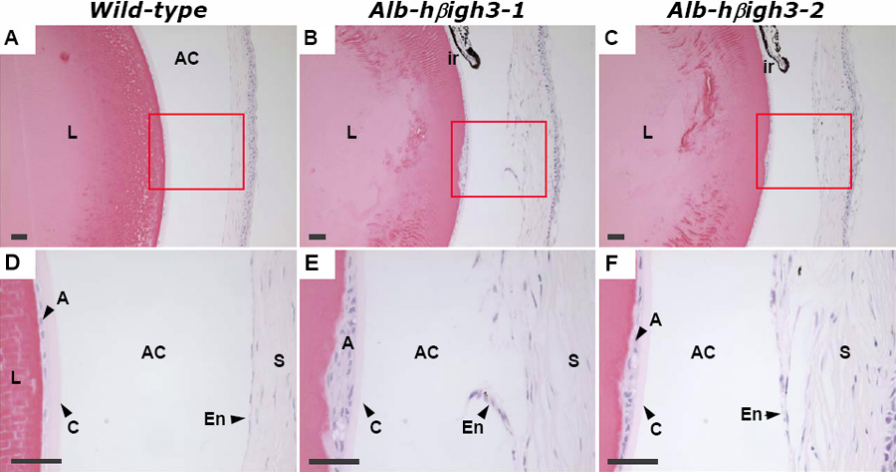
Histopathologic findings of normal eye in wild-type mouse and the defective eye in transgenic mouse at two months of age

**Figure 6:**
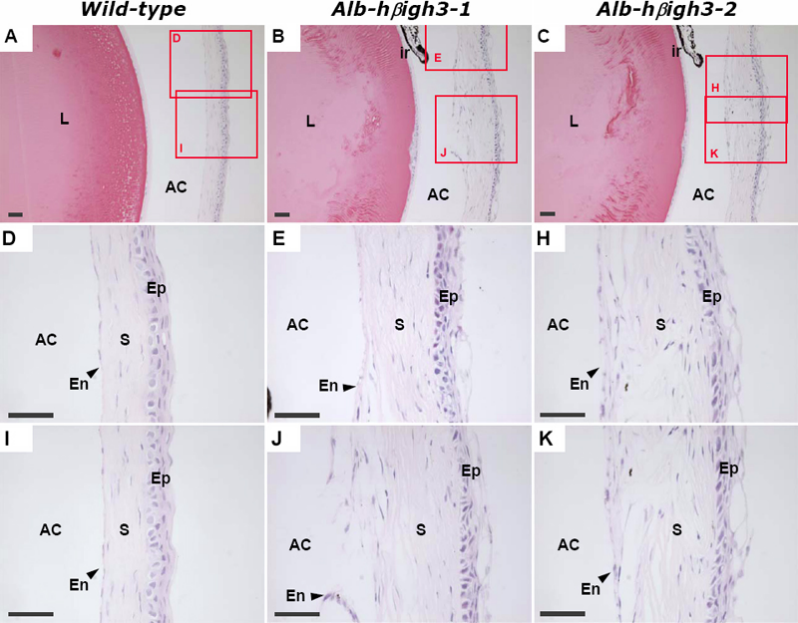
Histological examination of the defective eye in transgenic mouse compared to normal eye in wild-type mouse at two months of age

Ocular histology was analyzed in the eyes of wild-type and Alb-hßigh3 transgenic mice. Though the extent of the defect varied in each transgenic mouse, all mutant eyes had abnormalities at layers of the cornea, anterior chamber, and lens. In H&E stained sections, normal cornea was separated from the lens by a distinct endothelial layer and the corneal epithelium showed well defined, stratified squamous epithelium with a smooth surface (Figure 5A, Figure 6A). In comparison, transgenic mice with corneal opacity displayed an obvious abnormal cornea with an irregular corneal epithelium and an uneven corneal stroma, including disorganization of collagen layers in the defective area (Figure 6B,C). The defective eye also had a narrow anterior chamber and showed either partially formed or discontinued corneal endothelium (Figure 5B,C). Unlike the thin monolayer of lens epithelial cells in the lens' anterior surface of wild-type mice, the anterior region of the lens had abnormal multilayer cells in transgenic mice (Figure 5).

Staining was performed with H&E in each 4 μm thick paraffin section. **C** is the serial section of **B**. Images in row 2 are higher-magnification images corresponding to small red rectangles in row 1. The abnormal cornea of transgenic mice (**B**, **C**) had disorganized corneal stroma, disconnected corneal endothelium, and a narrow anterior chamber compared to wild-type (**A**). The anterior surface of the lens had multilayer epithelial cells in transgenic mice. The scale bars are equal to 50 μm. Abbreviations: L, lens; C, lens capsule; A, anterior epithelium of lens; AC, anterior chamber; ir, iris; S, corneal stroma; En, corneal endothelium.

Staining was performed with H&E in each 4 μm thick paraffin sections. **C** is the serial section of **B**. Images in rows 2 and 3 are higher-magnification images of the cornea corresponding to the two red rectangles in column 1. The abnormal cornea of transgenic mice (**B**, **C**) had disorganized corneal stroma and disconnected Descemet's membrane and endothelial cell layer compared to wild-type (**A**). The scale bars are equal to 50 μm. Abbreviations: L, Lens; ir, iris; AC, anterior chamber; Ep, corneal epithelium; S, corneal stroma; En, corneal endothelium.

**Figure 7:**
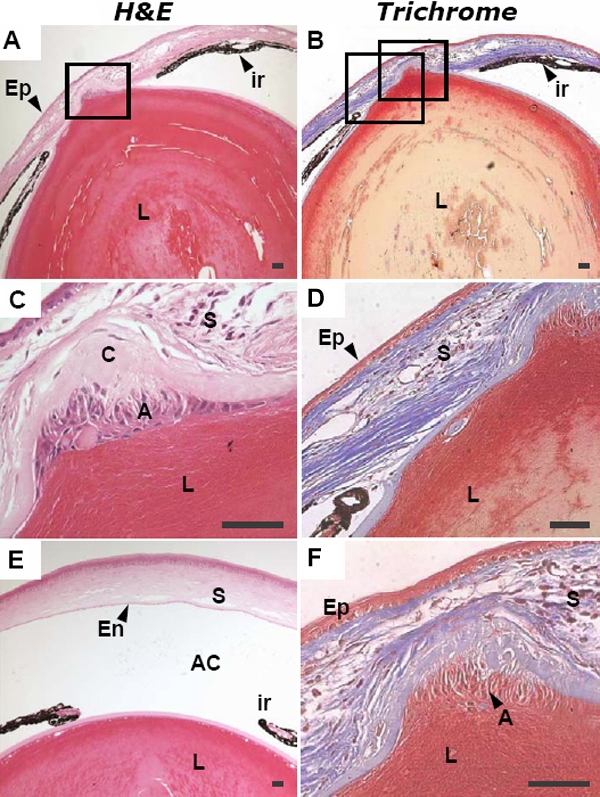
Histologic analysis of the anterior segment in a seven-month-old Alb-hßigh3 transgenic mouse

In another transgenic mouse that showed more serious deformity of the eye (Figure 7), the separation between lens and cornea was not complete. The anterior portion of the lens attached to the posterior surface of the cornea and the anterior chamber was obliterated with the iris adherent to the posterior surface of the cornea. Proliferated lens epithelial cells under the lens capsule were observed in the attached portion between the protruded lens and cornea. Thin corneal epithelium and disordered corneal stroma were also observed in the defective eye of the transgenic mouse. As shown in the wild-type mouse of Figure 5, the cornea was separated from the lens by a distinct endothelial layer; this was also the case in the seven-month-old wild-type mouse (Figure 7E).

Staining was performed with H&E (**A**, **C**, and **E**) and Trichrome (**B**, **D**, and **F**) in each 4 μm thick paraffin section. Images in rows 2 and 3 are higher-magnification images corresponding to the small rectangles in column 1. Separation between lens and cornea was not complete in the defective eye of the Alb-hßigh3 transgenic mouse. The attached portion between the protruded lens and cornea showed proliferated lens epithelial cell under the lens capsule. The terminal part of the iris was attached to the posterior surface of the cornea and there was no space for the anterior chamber. **E** shows the normal eye of a seven-month-old wild-type mouse. The scale bars are equal to 50 μm. Abbreviations: L, Lens; C, lens capsule; A, anterior epithelium of lens; ir, iris; Ep, corneal epithelium; S, corneal stroma; En, corneal endothelium.

## Discussion

A transforming growth factor-β (TGF-β)-induced extracellular matrix (ECM) protein, βigh3, has been considered a major component of abnormal extracellular deposits in the cornea and its mutations and responsible for 5q31-linked human autosomal dominant corneal dystrophies (CDs) [8]. Immunohistochemical studies show that βigh3 is strongly stained in pathologic deposits in all mutant βigh3-related corneal dystrophies and that, above all, high levels of normal βigh3 are detected in scarring cornea [18] as if TGF-β is present on the edge of wound healing in the cornea [19]. These data suggest that βigh3 as well as TGF-β would be key molecules in the pathogenesis of corneal opacification. In this study, we investigated whether βigh3 is essential to ocular development in vivo and whether it is pathologically important to corneal disorganization.

First, to study the function of βigh3 in eye, it is necessary to discover suitable methods or animal models that express ectopic or mutant βigh3. Many researchers have tried direct injections or eye drops of plasmid DNA or infection with an adenovirus vector in the eye. However, the difficulty of gene transfer into the cornea or the eye without inflammation prevents the study of certain protein's roles in these tissues [20,21]. Therefore, diverse methods to overcome this difficulty have been studied and reported. Several mouse model systems have been developed to observe the function of TGF-β, which is an inducer of βigh3 and has a critical role in the development of normal cornea [22,23]. Even though overexpression of TGF-β1 driven by the lens-specific αA-crystallin promoter does not cause a corneal phenotype in the embryonic mouse eye, cataract formation is observed in adult mice with ocular defects including corneal opacity and structural changes in the iris and ciliary body [24]. Transgenic overexpression of TGF-β1 by lens-specific chicken βB1-crystallin promoter also revealed severe disruption of corneal and anterior chamber development from mouse embryonic day 13.5 [25]. These results suggest not only the importance of precise quantitative control of TGF-β but also of good transgenic model systems to study the role of TGF-β in eye development. However, TGF-β overexpression regulated by these specific promoters may cause an artificial and excessive phenotype that is restricted to a specific time and tissue in lens development.

Sakamoto et al. [26] constructed an adenoviral vector expressing a soluble TGF-β receptor fused to the Fc portion of human IgG and injected this construct into the skeletal muscle of mice. The soluble TGF-β receptor produced in the muscle reached the cornea by means of normal blood circulation, sequestered local TGF-β, and acted in the cornea but not in the lens. Based on this idea, we generated transgenic mice that overexpressed human βigh3 (hßigh3) in blood through the liver-specific albumin (Alb) enhancer/promoter. Albumin, which is a major secretory protein of the liver, is induced in fetal liver and its expression is maintained in the adult liver [27]. Since serum albumin is synthesized particularly by hepatocytes, the albumin promoter/enhancer is activated during liver development. Previously, it had also been reported that the most abundant water-soluble protein in the human cornea, serum albumin, can diffuse from peripheral blood vessels around the cornea [28,29]. βigh3 is a secretory protein and hßigh3 ectopically expressed in liver under the control of Alb promoter was able to secrete into blood. Eventually, this secreted protein reached the cornea via blood vessels surrounding the limbal area of the cornea where it affected the anterior segment. In this model system, we can exclude possible artificial outcomes in the lens caused by using αA- or βB1-crystallin lens-specific promoters and explain the role of βigh3, which causes corneal diseases in human.

An exogenous transgene in the transgenic mouse model should be clearly distinct from an endogenous gene. For this, many researchers use specific marker genes or the same gene from different species. By using the human βigh3 transgene, exogenous transgene is easily distinguished from endogenous mouse βigh3. Since the nucleotide sequence of human βigh3 has high identity with mouse βigh3 and the amino acid sequence also has more than 91% identity with mouse, there were no undesirable phenotypes caused by using a transgene from a different species.

Here, we demonstrated that the Alb-hßigh3 transgenic mouse had a serious failure in anterior segment development including an irregularity of the corneal epithelium, disorganization in collagen layer of the corneal stroma, and discontinuity of corneal endothelium following corneal opacity. Thickened corneal abnormality due to endothelial disruption was observed in the cornea of Alb-hßigh3 transgenic mice with corneal opacity. Moreover, the attachment of the iris or lens to the cornea in some transgenic mice indicated that overexpression of βigh3 resulted in incomplete central migration of neural crest cells during ocular development. Cataract formation was also examined in defective eyes of Alb-hßigh3 transgenic mice. In a previous paper, we showed that βigh3 expression greatly increased in lens epithelial cells from patients with anterior polar cataracts and in human lens epithelial cells treated with TGF-β [30]. Thus, cataract formation in transgenic mice was due to overexpression of βigh3 in their affected eyes. The bilateral phenotype with cataract in Alb-hßigh3 transgenic mice was very similar to the clinicopathologic findings of Peters' anomaly that is a kind of anterior mesenchymal dysgenesis in human developmental anomalies. Most cases of Peters' anomaly are bilateral with lens abnormality and variable severity [31,32]. However, mild unilateral cases are often observed in patients who do not have cataract. Additionally, even though hßigh3 was overexpressed in the entire mouse body through the blood stream, the effect was observed only in the eye. This result corresponds exactly with the observation that mutant βigh3 in humans causes only corneal dystrophy in the eye and no other deformities in other organs [33].

The anterior segment of the vertebrate eye is structurally defined by the cornea, iris, ciliary body, and lens [34]. Various anterior segment mesenchymal dysgenesis have been reported in humans and animals. In particular, anterior segment disorders in humans include autosomal dominant iridogoniodysgenesis anomaly, family glaucoma with goniodysgenesis, congenital endothelial dystrophy, and aniridia [35-38]. Although coordinated interactions between different cell types in these disorders are considered essential for proper spatial positioning and differentiation, the requisite intercellular signals and the proper animal model to verify these signals have not been well defined. One human anterior segment disorder, Peters' anomaly, is characterized by congenital corneal opacity with defects in the cornea [39]. By histological examination, Peters' anomaly in humans shows dense corneal opacity, iridocorneal adhesions, and occasional lens abnormality with direct adhesion to the posterior corneal surface. In addition, human Peters' anomaly accounts for a thickened cornea due to endothelial layer disruption. The histological views of Alb-hßigh3 transgenic mice with corneal opacity are comparable to phenotypes observed in human Peters' anomaly. Likewise, a mouse model of fetal alcohol syndrome (FAS) has been reported to exhibit similar defects to Peters' anomaly with malformations in Descemet's membrane and corneal endothelium as well as delayed or failed separation of the lens [40]. Accordingly, Alb-hßigh3 transgenic mice will be a practical model to study these eye disorders.

Though we have not examined the signaling pathway that is altered by overexpression of βigh3, these results sufficiently explain that βigh3 is involved in anterior segment morphogenesis and must be properly expressed for normal anterior segment development. This suggests that βigh3 may play an important role in the corneolenticular adhesion and the normal development of the cornea during ocular morphogenesis. Therefore, βigh3 may play a role in the normal formation of the anterior segment. Taken together, this study shows that βigh3 expression must be critically maintained during ocular development to avoid severe deformity, especially in the cornea. The Alb-hßigh3 transgenic mice described in this study also provide a useful animal model for the study of anterior segment dysgenesis and of the control of proliferation and differentiation of neural crest-originated cells.
